# Social isolation in adults with cancer: An evolutionary concept analysis

**DOI:** 10.3389/fpsyg.2022.973640

**Published:** 2022-10-03

**Authors:** Yanjing Liang, Guihua Hao, Mei Wu, Lili Hou

**Affiliations:** ^1^School of Nursing, Shanghai Jiao Tong University, Shanghai, China; ^2^Nursing Department, Shanghai Ninth People’s Hospital, Shanghai Jiao Tong University School of Medicine, Shanghai, China

**Keywords:** social isolation, cancer, psychological nursing, psychosocial behavior, concept analysis

## Abstract

**Background:**

In extant literature, the concept of social isolation has been explored primarily in the context of older adults. However, people with cancer may also experience social isolation, and there is a need for increased clarity regarding this phenomenon in this population.

**Objective:**

To conceptualize social isolation in adult cancer care.

**Methods:**

PubMed, Web of Science, PsycINFO, CINAHL, China National Knowledge Infrastructure, Wanfang Data, and the Chinese Biomedical Literature Database were systematically searched using the key terms “cancer,” “social isolation,” “social alienation,” and “social exclusion” for studies (from the earliest date available to June 2022). The main disciplines involved were psychology, nursing, medicine, and public health. Rodgers’ evolutionary concept analysis was employed to clarify the antecedents, attributes, and consequences of social isolation in adults with cancer.

**Results:**

A total of 60 eligible articles were reviewed entirely and the main findings were categorized into antecedents, attributes, and consequences. The antecedents of social isolation were classified into six categories: cancer-related physiological changes, cognitive beliefs, psychological wellbeing, unsatisfactory social supports or relationships, restrictions associated with receiving treatments, and social-level barriers. Attributes were characterized according to behavior or social avoidance and negative affective experiences, while consequences were attributed to low therapeutic compliance, poor health conditions and mental health problems, and low quality of life. White’s heuristic model is a potential theoretical context applicable to social isolation in adults with cancer.

**Conclusion:**

This concept analysis provides a basis for developing multidimensional assessment tools and measures to alleviate social isolation in adults with cancer, a complex and varied phenomenon. However, while this review contributes to the current knowledge on social isolation in people with cancer, studies should further investigate the relationships among attributes associated with social isolation.

## Introduction

Globally, over 19 million individuals are diagnosed with cancer annually (Sung et al., 2021). Cancer diagnosis and treatment can cause severe adversity, resulting in varying psychological distress levels (Sender et al., 2020). However, with advances in healthcare, survival rates are also higher, and there is now increased advocacy to enhance the quality of survivorship care. Consequently, treatment goals include improving patients’ quality of life, symptom management, and psychosocial effects (Bray et al., 2018; Nekhlyudov et al., 2019). Despite this, limited attention has been paid to severe psychosocial issues, among which social isolation is one.

The term “social isolation” was introduced in Berkman and Syme’s (1979) seminal study. Initially, it referred to individuals who lacked social networks or had limited social supports. Thereafter, it was developed concurrently across multiple disciplines including public health, medicine, and nursing (Nicholson, 2009). In public health literature, the definition of social isolation evolved from “…irreversible loss of social bonds” (Berkman, 1983) to “lack of attachments within a person’s social network” (LaVeist et al., 1997). In the context of nursing, the subjective feeling of the unmet needs for social connections or supports and experiences of loneliness have been emphasized (Lien-Gieschen, 1993; Biordi, 1998; Ackley and Ladwig, 2004), while medicine views it as an objective indicator of social contact (Havens et al., 2004). The final definition of this concept incorporates both subjective and objective measures. Specifically, social isolation is characterized by minimal contact with other people and limited involvement in community affairs (Iredell et al., 2004).

The aforementioned definitions of social isolation have generally been developed with reference to older adults. Thus, an oncology-specific multidimensional definition of social isolation is yet to be clarified. The experience of social isolation and its associated attributes may differ among various age groups and by health conditions. Furthermore, social isolation is a phenomenon associated with severe health consequences and has been expanded to cancer. Specifically, in the context of oncology, social isolation has been analyzed primarily in patients with breast, prostate, head and neck, and lung cancers (Ettridge et al., 2018; Ashi et al., 2020; Dornan et al., 2021; Kudjawu and Agyeman-Yeboah, 2021).

Several studies underscore the impact of social isolation. Research indicates that social isolation is linked with the increased risk of tumor recurrence and mortality among patients with breast cancer (Kroenke et al., 2017). Friedler et al. (2015) summarized several studies on the pathophysiological mechanisms of social isolation, revealing that it affects the immune system, autonomic nervous system, and neuroendocrine axis. Moreover, long-term social isolation may increase the risk of mental illness (Yuan et al., 2020), reduce patient compliance with treatment, and increase cancer care costs (de Souza et al., 2017). Despite a lack of in-depth explorations, there is increasing evidence to suggest that social isolation could impact health in adults with cancer. This may be attributed to the different implications of social isolation between adults with cancer and older adults. The concept of social isolation in adults with cancer remains unclear. Therefore, this study aimed to clarify the meaning of social isolation in adults with cancer and examine its antecedents, attributes, and consequences by conducting a concept analysis.

## Materials and methods

### Identifying a concept

Rodgers’ concept analysis method is an inductive process (Rodgers, 2000). In Rodgers’ view, concepts evolve and are shaped by the context in which they are used. Therefore, they are constantly developed and redefined. There have been studies on social isolation across various fields. Furthermore, the meaning of the associated terms has been established over the years. Thus, we applied Rodgers’ evolutionary concept analysis method and reviewed the literature on social isolation focusing on people with cancer to understand social isolation in the cancer context (Table 1; Rodgers, 2000).

**Table 1:** Steps for Rodgers’ evolutionary concept analysis.

Step	Description	
1	Identify a concept and its surrogate terms.	
2	Determine and select a suitable data collection scope.	
3	Data collection: Concept attributes; Contextual basis including temporal, sociocultural, and interdisciplinary variables.
4	Data analysis.	
5	If necessary, provide examples supporting the concept.
6	Identify hypotheses and applications for the concept’s future development.

### Sources of data

We conducted systematic electronic searches (from the earliest date available to June 2022) of PubMed, Web of Science, PsycINFO, CINAHL, China National Knowledge Infrastructure, Wanfang Data, and the Chinese Biomedical Literature Database using combinations of the terms “cancer,” “social isolation,” “social alienation,” and “social exclusion” in the abstract or title. The inclusion criteria were articles that were published in English or Chinese; had undergone peer review (excluding dissertations, theses, or comments); and underscored social isolation in people with cancer, specifically concepts, antecedents, attributes, and consequences. Searches were conducted without any time and discipline constraints to ensure the identification of as many relevant articles as possible and to provide an overview of the concept’s use over time. The exclusion criteria were published abstracts of studies that did not contain specific information on social isolation and studies conducted on non-cancer populations.

**Figure 1:**
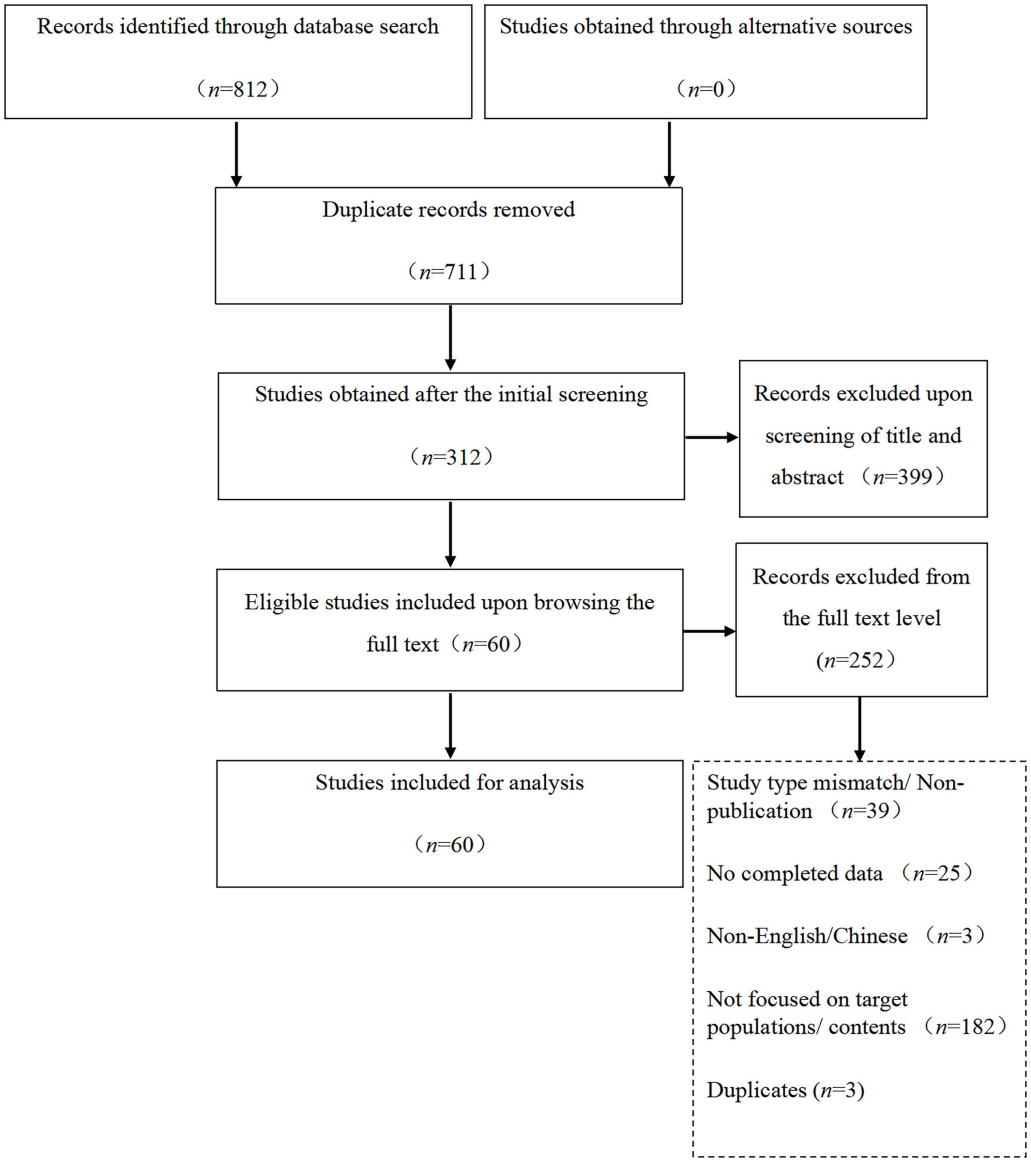
Flow chart of the study screening process. The studies obtained from each database are as follows: PubMed (*n* = 16), Web of Science (*n* = 5), PsycINFO (*n* = 22), CINAHL (*n* = 12), China National Knowledge Infrastructure (*n* = 3), Wanfang Data (*n* = 2), and the Chinese Biomedical Literature Database (*n* = 0).

The original search resulted in 711 articles after deleting duplicates using the NoteExpress 3.5.0.9054 software (Figure 1). Upon performing further screening based on the exclusion criteria by reading the titles and abstracts of the articles, a sample size of 312 was obtained. There was no consensus on the criteria for evaluation of the data sources included in the concept analysis; only articles that mentioned social isolation in people with cancer were included. Duplicate studies that were not recognized by the software were manually deleted. Studies with insufficient information regarding the characteristics of social isolation in people with cancer, making attribute identification difficult, were also removed. This resulted in the removal of 252 of the 312 studies. Rodgers (2000) recommends that the total number of references per discipline included in a study should be approximately 20% of the total. This was not feasible in the current study. To gain a deeper insight into social isolation, the remaining 60 selected studies were included in this concept analysis. Finally, the disciplines involved were psychology (*n* = 12), nursing (*n* = 24), medicine (*n* = 19), public health (*n* = 3), medical informatics (*n* = 1), and epidemiology (*n* = 1).

### Data collection and management

Based on Rodgers’ method, the articles were studied for the identification of surrogate terms and related concepts. Furthermore, they were reviewed for systematic data collection and management in terms of attributes, antecedents, and consequences of the concept. The aforementioned information was extracted and tabulated. Among the 60 articles reviewed, an explicit definition of social isolation was rare. Thus, not all articles were extracted for their attributes of the concept. The data sources were reviewed by two of the researchers and checked by a third (Zhu et al., 2018). The matrix was developed by YL and MW.

### Data analysis

The included articles were coded according to antecedents, attributes, consequences, surrogate terms, contextual basis, and theoretical and practical definitions. The coding was conducted using the standard procedure of thematic analysis underscored in Rodgers’ evolutionary method (Rodgers, 2000). Independently, two reviewers partially completed the work before discussing it with a third to reach a cohesive and comprehensive consensus to minimize bias as much as possible. Finally, the review team developed the conceptual definition of social isolation in people with cancer (Zhu et al., 2018).

## Results

### Study characteristics

The studies included in this analysis were published between 1980 and 2022; 55 were in English and five were in Chinese. Most employed cross-sectional and qualitative research designs. Patients with various types of cancer such as those affecting the lung, breast, head and neck, and prostate were included. The sample sizes ranged from three to 25,382. Only some studies included all the definitions, antecedents, attributes, and consequences of social isolation.

### Surrogate terms and related concepts

Multiple terms have been used interchangeably with social isolation. For example, social alienation has been used in place of social isolation (Yang et al., 2021). While the term “social exclusion” is distinct from “social isolation,” it has been used to describe social isolation in some studies (Dahill et al., 2020). There are two main concepts—alienation and loneliness—that need to be differentiated from social isolation. According to Seeman (1959), alienation encompasses powerlessness, isolation, normlessness, self-estrangement, and meaninglessness. Thus, isolation is only one psychological state of alienation. Some researchers use “loneliness” to express “social isolation.” However, we did not use “loneliness” as a surrogate term in our search; using “social isolation” and “loneliness” interchangeably can be confusing, as we found loneliness to be an attribute of social isolation (Raque-Bogdan, 2019; Raque-Bogdan et al., 2019).

### Social isolation definitions used in different cancer populations

Definitions of social isolation in adults with cancer have been presented in several studies with varying emphasis. Reduced social networks and loneliness are the most prevalent aspects of these definitions. Changing social health contexts, as influenced by factors such as coronavirus disease 2019 (COVID-19), have led to a spate of studies related to social isolation (Table 2).

**Table 2:** Samples of social isolation definitions applied in studies included in the analysis.

Author (year)	Definitions of social isolation used in the article
Kirtane et al. (2022)	Physical and emotional social isolation is experienced during special circumstances (COVID-19).
Takemura et al. (2021)	A social relationship shortfall can be quantified by social network size, diversity, or frequency of contacts.
Liu B et al. (2021) and Liu Y et al. (2021)	People are isolated from interpersonal interaction and relationships. It is considered social isolation if people have limited social contact or communication and limited participation in social activities or meetings with friends.
Kudjawu and Agyeman-Yeboah (2021)	Social isolation refers to avoidance behaviors and feelings of isolation while dealing with breast cancer.
Liu B et al. (2021) and Liu Y et al. (2021)	Social isolation refers to an individual’s unsatisfied social desire and failure to interact well with the outside world, accompanied by negative emotions such as loneliness and helplessness and negative behaviors such as apathy and rejection.
Morreale et al. (2020)	Feelings of being avoided, excluded, detached, disconnected, or not being known to others.
Yuan et al. (2020)	In traditional sociological terminology, social isolation entails a sense of anomie, which includes feelings of loneliness, inequality with others, and uselessness.
Wang et al. (2020)	Social isolation refers to the phenomenon that an individual is automatically isolated from other people and society when they are treated negatively by the world during social interaction, resulting in negative emotions such as loneliness and helplessness, and showing negative behaviors such as avoidance and rejection.
Zhu et al. (2019)	Social isolation refers to people believing that their relationships are insufficient to meet the quality and quantity of their social needs.
Raque-Bogdan et al. (2019)	The term “survivor loneliness” is used to represent the social isolation that may occur after cancer treatment. On an intrapersonal level, survivors described feeling alone as a result of acting inauthentically in relationships, feeling out of control of their bodies after the treatment, and feeling alone in their experience.
van Roij et al. (2019)	Social isolation refers to an abnormal life with an abnormal social network.
Carduff et al. (2018)	Inability to socialize or maintain existing relationships or develop new ones.
Parton et al. (2017)	According to some women, social isolation refers to being in a life stage that is not consistent with the life stages of their peers.
Biagioli et al. (2017)	Protective isolation is used to keep patients away from infections that may negatively affect them.
Hinzey et al. (2016)	A person’s level of perceived social isolation (colloquially, loneliness) can be measured objectively based on criteria such as the size of their social network and number/frequency of interactions with others, or subjectively based on how isolated they perceive themselves to be.
Jeong et al. (2016)	Social interactions provide a means of measuring social isolation objectively.
Kroenke et al. (2013)	Small social networks are considered social isolation.
Fu et al. (2013)	Intentionally avoiding social or public appearances or contact. The five components that make up a social network are a spouse or intimate partner, the number of first-degree female relatives (living mother, number of biological daughters, number of full sisters), friendship relationships, religious or social ties, and community involvement.
Lee et al. (2011)	Social isolation, which refers to rejecting human contact, is practiced by patients as a way of protecting themselves.
Bennett et al. (2006)	Social isolation refers to physical and emotional isolation. It may have also been a means of protecting the self, friends, and family members. It is the result of attempting to appear “normal” and concealing one’s true emotions, along with avoidance behaviors.
Hagedoorn and Molleman (2006)	Social isolation is the result of other people avoiding cancer survivors, and the survivors isolate themselves from their families and friends because of concerns about appearances and reactions.
Høybye et al. (2005)	It isolates them from loved ones and from the social world they once enjoyed.

### Attributes

A concept is defined by distinct meanings, referred to as attributes. Attributes comprise a set of characteristics that can be used to categorize similar situations related to a certain concept (Rodgers, 2000). During the analysis, two attributes underscored by succinct generalizations of social isolation in people with cancer emerged. These attributes recurred in the description of social isolation in data sources, and included behaviors and states of social avoidance as well as negative affective experiences. Studies supporting each attribute are shown in Table 3. Notably, there is a significant difference between external social isolation and perceived or internal social isolation. However, some patients experiencing the former (i.e., behaviors and states of social isolation) may not experience negative affective experiences. It is imprudent to classify all the behaviors and states of social avoidance simply as social isolation and ignore emotional isolation.

**Table 3:** Overview of the characteristics and attributes of social isolation defined in the studies included in the concept analysis.

Author (year)	Country	Oncology population	Discipline	Design	Sample	Attributes: behaviors and states of social avoidance	Attributes: negative affective experiences
Kirtane et al. (2022)	United States	Head and neck cancer	Medicine	Qualitative study	20	Restricted social network	Distress
Takemura et al. (2021)	Japan	Lung cancer	Medicine	Prospective cohort study	264	Limitations with social networks (size, diversity, or frequency of contacts)	Feelings of loneliness
Liu B et al. (2021) and Liu Y et al. (2021)	China	Breast cancer	Medicine	Cross-sectional study	389	Restricted social network (contact, activity, communication)	
He et al. (2021)	China	Rectal cancer	Nursing	Qualitative study	18	Avoidant behaviors (protection from identifying with any form of cancer) Concealing their real self	
Kudjawu and Agyeman-Yeboah (2021)	Ghana	Breast cancer	Medicine	Qualitative study	8	Restricted social network (contact, activity, communication)	
Liu B et al. (2021) and Liu Y et al. (2021)	China	Lung cancer	Nursing	Cross-sectional study	288	Limitations with social networks Avoidant behaviors	Feelings of loneliness Feelings of helplessness
Yuan et al. (2020)	China	Head and neck cancer	Nursing	Cross-sectional study	230		Feelings of loneliness Feelings of uselessness
Wang et al. (2020)	China	Breast cancer	Nursing	Cross-sectional study	228	Limitations with social network Avoidant behaviors	Feelings of loneliness Feeling of helplessness
Zhu et al. (2019)	United States	Prostate cancer	Medical informatics	Retrospective study	3,138	Limitations with social networks Considering relationships insufficient to meet social needs (lacking companionship)	Feelings of loneliness Feelings of social exclusion
Raque-Bogdan et al. (2019)	United States	General cancer	Psychology	Review		Concealing their real selves (including hiding cancer facts and feelings of loneliness)	Feelings of loneliness A feeling of a lack of control over one’s body
van Roij et al. (2019)	Netherlands	General cancer	Medicine	Qualitative study	18	Abnormal social network Abnormal life	
Ettridge et al. (2018)	Australia	Prostate cancer	Nursing	Qualitative study	20	Self-isolating or concealing oneself	Feelings of loneliness Low self-esteem
De Blasi et al. (2018)	France	General cancer	Medicine	Qualitative study	3	Relinquishing former social roles	Feelings of social exclusion (working environment)
Carduff et al. (2018)	United Kingdom	Bowel cancer	Nursing	Qualitative study	16	Limitations with social networks (inability to maintain old and develop new relationships)	
Iannarino (2018)	United States	General cancer	Medicine	Qualitative study	3	Limitations with social network	
Puigpinós-Riera et al. (2018)	Spain	Breast cancer	Medicine	Mixed cohort studies	2,235	Limitations with social networks (size, frequency of contacts)	Distress
Simonelli and Otto (2017)	United Kingdom	Gynecological cancer	Psychology	Book		Limitations with social networks	Low self-esteem Feelings of social exclusion (working environment)
Parton et al. (2017)	United Kingdom	Cancer in women	Psychology	Mixed-methods study	695		Feelings of being in a life stage that is not consistent with peers’ life stages
Biagioli et al. (2017)	Italy	Hematologic malignancies	Nursing	Qualitative study	9	Protective isolation	Feelings of loneliness
Hinzey et al. (2016)	United States	Breast cancer	Medicine	Review		Limitations with social networks (size, diversity, or frequency of contacts)	Feelings of loneliness
Jeong et al. (2016)	Korea	Lung cancer	Nursing	Systemic review		Abnormal social interactions Exclusion from social support groups Lack of advocacy Response to stigmatization	Feelings of loneliness
Fu et al. (2013)	Australia	Breast cancer	Psychology	Systemic review		Not going out Avoiding contact with others	
Kroenke et al. (2013)	Oakland	Breast cancer	Epidemiology	Cohort studies	2,264	Limitations with social networks (size)	
Lee et al. (2011)	Korea	Neutropenic cancer	Nursing	Systematic review		Limitations with social networks	Concealing real emotions Feelings of powerless
Bennett et al. (2006)	United Kingdom	Breast cancer	Nursing	Qualitative study	8	Avoidant behaviors (protection from identifying with any form of cancer) Concealing their real selves	Feelings of loneliness
Hagedoorn and Molleman (2006)	Netherlands	Head and neck cancer	Public health	Cross-sectional study	76	Avoidant behaviors (both active and passive isolation)	
Blinderman and Cherny (2005)	Israel	General cancer	Psychology	Qualitative study	40	Limitations with social networks Avoiding contact with others	
Høybye et al. (2005)	Denmark	Breast cancer	Medicine	Qualitative study	39		Feelings of loneliness
Noyes et al. (1990)	United States	Solid tumors	Psychology	Cross-sectional study	438	Limitations with social networks (family, friends)	
McGeough et al. (1980)	United States	Lung cancer	Nursing	Cross-sectional study	22	Limitations with social networks (withdrawal from family and friends)	Feelings of loneliness

#### Behaviors and states of social avoidance

##### States of social avoidance

States of social avoidance include limitations regarding social networks, which are determined by the frequency of social contact, activity, communication, size, and diversity (Liu B et al., 2021; Liu Y et al., 2021; Takemura et al., 2021). Discontinuing work after cancer diagnosis or treatment and relinquishing social roles are common for patients, and exacerbate feelings of isolation (De Blasi et al., 2018). To some extent, the choices of discontinuing work or relinquishing social roles belong to the social avoidance behavior attribute.

##### Social avoidance behaviors

Some cancer survivors choose to self-isolate (Ettridge et al., 2018; He et al., 2021). They are often reluctant to communicate with others, specifically concerning details of their illness. Social avoidance behaviors are more common among patients with prostate and rectal cancer (Ettridge et al., 2018; Zhu et al., 2019; He et al., 2021). Social isolation can be a passive choice induced by the inability to socialize like before due to medical conditions, surgery, and treatment effects. Notably, some cancer survivors conceal their identities and emotions (including hiding cancer-related information and feelings of loneliness) to protect themselves or their loved ones. This paradoxical behavior, aiming to eliminate negative emotions such as loneliness while hiding critical health-related facts from medical staff, patients, and their loved ones, requires increased prioritization (Raque-Bogdan, 2019).

#### Negative affective experiences

Loneliness has been reported as a negative effect among patients for several reasons. Cancer survivors experience distress, as they cannot speak about their illness with anyone except their intimate partners and medical specialists (Ettridge et al., 2018; Zhu et al., 2019).

Furthermore, low self-esteem, generally linked to self-discrimination, has been identified as a negative affective experience. Some patients may experience negative self-perception or self-denial due to cancer-related physiological changes, and thus avoid contact with people around them (Blinderman and Cherny, 2005; Ettridge et al., 2018). Behaviors and states of social avoidance and negative affective experiences are closely related. Another manifestation of low self-esteem is the fear of being humiliated in front of others (Johansson et al., 2005).

Feelings of social exclusion are common negative experiences resulting from the incapability to perform family or work roles owing to illness and result in survivors feeling excluded from society (Rhoten et al., 2013; Simonelli and Otto, 2017; De Blasi et al., 2018; Puigpinós-Riera et al., 2018). For some women and young adults, the feeling of being in a life stage that is not consistent with peers is also an attribute of social isolation (Parton et al., 2017; De Blasi et al., 2018; Iannarino, 2018).

### Antecedents

Antecedents are events or phenomena that precede the concept (Rodgers, 2000). Six antecedents of social isolation were identified in adults with cancer. These included cancer-related physiological changes, cognitive beliefs, psychological wellbeing, unsatisfactory social supports or relationships, restrictions on receiving treatments, and social-level barriers.

#### Cancer-related physiological changes

Physiological change is the most common antecedent of social isolation among cancer survivors. Cancer survivors may experience physical and functional impairments caused by their illness and treatment, thus resulting in limited social integration (Noyes et al., 1990; Sandén and Hydén, 2002; Johansson et al., 2005; Lund-Nielsen et al., 2005; Bennett et al., 2006; Simonelli and Otto, 2017; De Blasi et al., 2018; Abdollahimohammad et al., 2019; Borgi et al., 2020; Stolley et al., 2020; Yuan et al., 2020; He et al., 2021; Kudjawu and Agyeman-Yeboah, 2021; Tsui and Huang, 2021). For instance, the complications associated with head and neck cancer, such as disfigurement, dysphagia, and speech impairment, can impair social functioning among survivors, exacerbating social isolation (Rogers et al., 2016; Moore et al., 2018; Kirtane et al., 2022). For gastrointestinal cancer survivors, gastrointestinal-specific morbidities such as ostomy leakage and incontinence deter them from an active social life (Martopullo et al., 2020; He et al., 2021). Other cancers (e.g., prostate, breast, and gynecological cancer) can impact survivors’ sexual life, as they may consider it an embarrassing topic to raise with friends and partners. These individuals need support, such as from peer groups, to express their challenges and share experiences (Sandén and Hydén, 2002; Power and Hegarty, 2010; Campo et al., 2017; Ettridge et al., 2018; Iannarino, 2018). Thus, as their physical health deteriorates, cancer survivors become weaker, more dependent, and socially isolated, resulting in their inability to socialize as well as maintain and form relationships (Schapmire et al., 2012; Carduff et al., 2018).

#### Cognitive beliefs

Cognitive beliefs are also an important antecedent of social isolation. These refer to a set of habitual pattern recognitions that one is used to, which is “fundamental to a person’s world view” (Charles et al., 2006). According to White’s heuristic cognitive behavioral model (White, 2000), false beliefs may lead to negative behavior (e.g., social avoidance). Regarding patients with rectal cancer tolerating stomas, two extreme attitudes can arise and prevent them from engaging in social activities. Stomas are sometimes treated inappropriately by patients who think their stomas are infectious and should quarantine themselves, while other times patients overprotect them, believing that they are fragile and thus should not be exposed (He et al., 2021). Recently, cognitive and psychological influencing factors such as body image, which can lead to social avoidance in people with head and neck cancer, have become research hotspots in the tumor population (Rhoten et al., 2013). While the underlying mechanism of social isolation in people with cancer is unclear, several theories can improve the understanding of the process that leads to it. The fear-avoidance model (Newell, 1999) explains cancer-related changes from a cognitive-behavioral perspective. Social avoidance behavior among cancer survivors is caused by the fear of changing appearances and functions. Fear and avoidance are conditioned reflexes maintained by learned thinking that constantly reminds individuals of their flaws. Individuals who are unable to adapt to physical deficiencies often continue to avoid society. Regarding nursing practice, social isolation can be guided by assessing the potential factors through understanding the avoidance behaviors demonstrated by patients based on this model.

#### Psychological wellbeing

Psychological wellbeing is integral to holistic health. Psychological discomfort serves as an antecedent of social isolation in people with cancer. It has been suggested that social isolation correlates positively with symptoms of anxiety and depression in patients with breast cancer (Liu B et al., 2021; Liu Y et al., 2021). Studies have shown that people with cancer with impaired psychological status demonstrate higher social isolation levels (Im and Chee, 2021). Furthermore, anxiety and depressive symptoms have been identified as common predictors in patients with oral cancer who exhibit increased social isolation levels due to stigma (Yuan et al., 2020). A qualitative study conducted in Ireland found that cancer survivors experience persistent symptoms of fatigue, anxiety, depression, and pain after receiving treatment, which increases their social isolation and reduces their activity levels. Notably, the first year after treatment involves managing the transition to survivorship and returning to daily life; survivors often feel isolated during this time (Boland et al., 2019).

#### Unsatisfactory social supports or relationships

Unsatisfactory social support is also an important antecedent of social isolation among cancer survivors. First, it may arise from not having someone with whom to discuss cancer-related matters (D’Agostino and Edelstein, 2013; Ettridge et al., 2018; Zhu et al., 2019). According to cancer survivors, their loneliness results from external avoidance and misunderstandings from others and cancer’s long-lasting impact (Fu et al., 2013; Hinzey et al., 2016; Raque-Bogdan et al., 2019; Kirtane et al., 2022). Second, the need for special attention for individuals with several intersecting identities might be unnoticed. It has been underscored that having several intersecting identities (e.g., lesbianism) may perpetuate a sense of isolation among individuals after a cancer diagnosis owing to previous experiences with societal oppression or marginalization. Thus, it is imperative to analyze individuals’ underlying identities and life experiences before the illness to provide tailored social supports (Raque-Bogdan, 2019). Moreover, individuals who live alone and do not have family or friends nearby may be more likely to experience social isolation (Fu et al., 2013; Ettridge et al., 2018; Leung et al., 2021). Some cancer survivors have reported that support from physicians and family members declines upon receiving treatment. This often leaves them feeling powerless and isolated because they still require care and support (Power and Hegarty, 2010; Kirtane et al., 2022).

#### Restrictions associated with receiving treatments

Patients with neutropenic cancer are sometimes placed in protective isolation at the hospital, enhancing their sense of security during treatment. Patients often practice self-isolation by refusing to interact with people, both emotionally and physically, to protect both themselves and others. Specifically, these patients often feel powerless (Lee et al., 2011). Similarly, several patients with head and neck cancer required isolation for radiochemotherapy during the COVID-19 pandemic, which exacerbated social isolation (Kirtane et al., 2022).

#### Social-level barriers

Research has indicated that the public health events associated with COVID-19 social distancing measures imposed more loneliness and social isolation, which are both stress-inducing factors (Miaskowski et al., 2020; Kirtane et al., 2022). Several lung cancer survivors reported that they were excluded from social activities. Consequently, experiences of exclusion increased their reluctance to engage with society (Liu et al., 2016). In Syria, cancer is not sufficiently understood and accepted by the public, and people with cancer often fear that chemotherapy will prevent them from marrying owing to effects that may impact their social functioning (Nizamli et al., 2011).

### Consequences

Consequences refer to the results of corresponding events or phenomena (Rodgers, 2000). The literature suggests that social isolation is generally accompanied by three consequences, namely, low therapeutic compliance, poor health conditions and mental health status, and low quality of life.

The first consequence of social isolation is low therapeutic compliance. Healthcare professionals often emphasize treatment compliance to patients. However, perceived social isolation among patients with cancer negatively impacts their treatment compliance (de Souza et al., 2017; Iannarino, 2018; Martopullo et al., 2020; Stolley et al., 2020). The second consequence is poor health conditions and mental health status. A systematic review has indicated that social isolation and loneliness can increase mortality rates, regardless of their link to underlying medical conditions (Kroenke et al., 2013; Holt-Lunstad et al., 2015; Zhu et al., 2019). Furthermore, a high symptom burden is associated with loneliness and social isolation (Adams et al., 2018). Social isolation also has effects, as low emotional support and social isolation are clear risk factors for increased anxiety and depression (Puigpinós-Riera et al., 2018; Alcaraz et al., 2020). Furthermore, social isolation in people with cancer is a significant issue that could aggravate depressive symptoms and increase tumor activity owing to the decreased expression of brain-derived neurotrophic factors (Borgi et al., 2020). Moreover, it can potentially contribute to the development of breast cancer through hormonal, angiogenic, and inflammatory markers and mediators (Hinzey et al., 2016). Low or impaired quality of life is a major consequence of social isolation, and health-related quality of life may be negatively affected by social isolation (Simonelli and Otto, 2017; Iannarino, 2018; Dahill et al., 2020; Martopullo et al., 2020; Dornan et al., 2021; Leung et al., 2021; Takemura et al., 2021).

### Conceptual definition

Based on the attributes, antecedents, and consequences identified in this analysis, we propose the following definition of social isolation in people with cancer:

Social isolation in adults with cancer refers to individuals demonstrating behaviors and states of social avoidance (e.g., avoiding contact with others and concealing one’s emotions) and experiencing specific negative affective experiences (e.g., feelings of loneliness). Social isolation can result from cancer-related physiological changes, cognitive beliefs, psychological well-being, unsatisfactory social supports or relationships, restrictions associated with receiving treatments, and social-level barriers. Social isolation can result in low therapeutic compliance, poor health conditions, mental health problems, and low quality of life.

**Figure 2:**
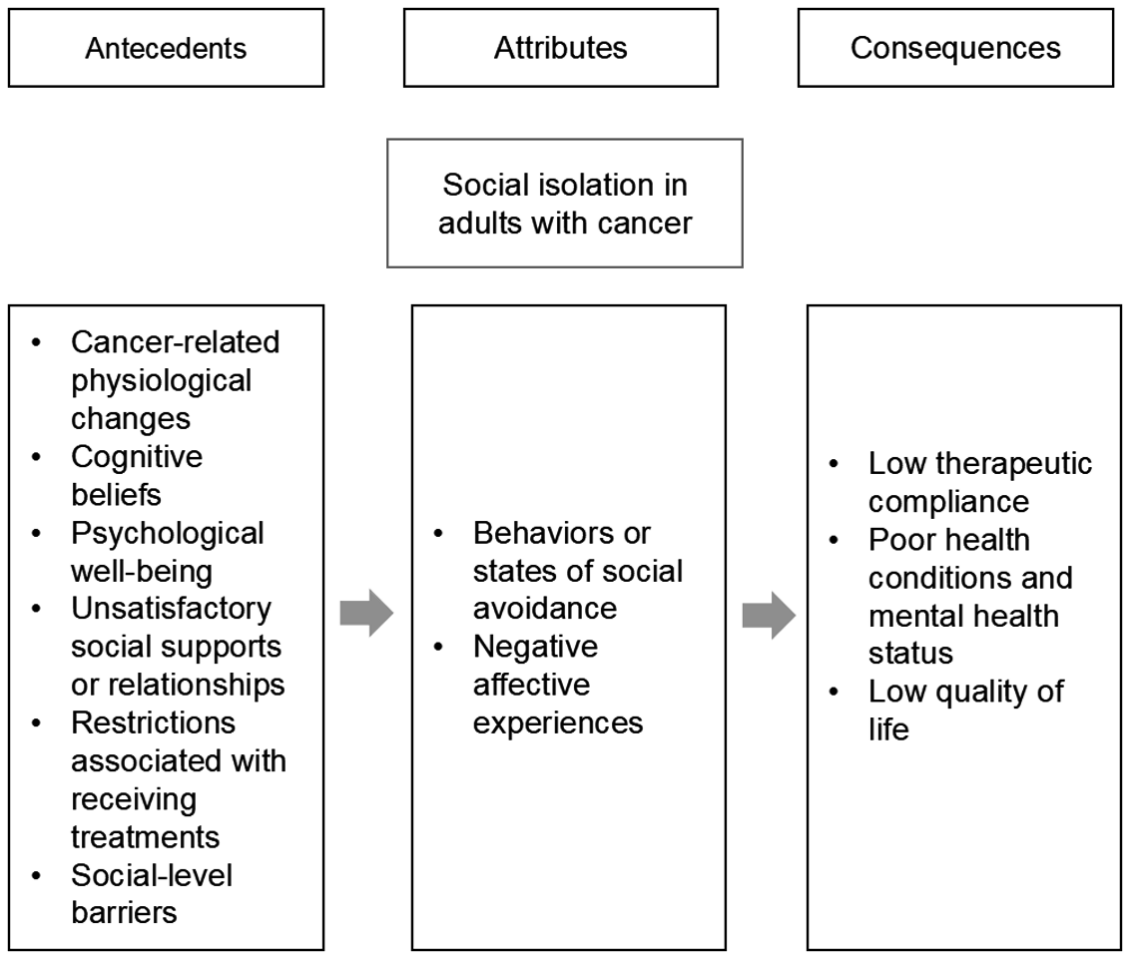
Model of social isolation concept in adults with cancer.

Figure 2 illustrates the negative antecedents, attributes, and consequences associated with social isolation in people with cancer.

## Discussion

### Significance of findings

Clear and precise conceptual systems play a significant role in the development of nursing knowledge (Tofthagen and Fagerstrom, 2010). Based on current knowledge, this is the first study to conceptualize social isolation in adults with cancer. Previous studies have only provided an in-depth analysis of issues related to social isolation in older adults (Nicholson, 2009). Furthermore, studies have underscored the effects of social isolation at the physiological level (Friedler et al., 2015). However, limited studies in this regard focus on people with cancer, who are also affected severely by social isolation. This may be why social isolation in people with cancer has not been explained adequately using the social isolation concepts from other populations. Therefore, it is necessary to clarify this concept in the cancer population. This concept analysis has established a set of attributes to define social isolation in adults living with cancer. Using standardized language developed using the concept analysis procedure, nurses and physicians can assess social isolation among cancer survivors and differentiate it from that among older adults and non-cancer survivors. It is crucial to recognize that social isolation in cancer survivors is a multidimensional experience influenced by several factors, particularly cancer-related physiological changes.

### Interpreting the results

In this analysis, the attributes of social isolation were classified into two categories, namely, behaviors and states of social avoidance and negative affective experiences. Insufficient social networks and feelings of loneliness were the most frequent characteristics of social isolation among the two categories. Table 3 indicates that only some of the included studies covered both categories. This may be owing to the varying cancer populations present in the included studies. Hence, the focus on social isolation in this analysis also differed.

No consensus was reached on the definition of social isolation in cancer populations. Compared to the behaviors and states of social avoidance, the negative affective experience attributes were more challenging to identify. Studies indicate that the latter attribute, which includes perceived social isolation and feelings of loneliness, is more harmful to mental health than the former (Liu B et al., 2021; Liu Y et al., 2021). Several patients were not aware that they were socially isolated despite having restricted social networks (Blinderman and Cherny, 2005; Tomaka et al., 2006). However, individuals can still feel lonely even if they are well-supported socially (Tomaka et al., 2006). Thus, it can be suggested that subjective or perceived social isolation warrants increased priority. During follow-up, nurses and physicians should pay close attention to the patient’s psychological changes and identify social isolation early.

### Identifying implications

White’s heuristic model stipulates that individuals will reshape their ideal self-image when their appearance and/or physical function are damaged (White, 2000). However, excessive expectations regarding their external image can lead to persistent and unpleasant emotions as well as social avoidance behaviors. Thus, people living with cancer may rebuild their ideal self-image after perceiving changes in appearance and physical function. However, owing to stigma, among other reasons, they could demonstrate compensatory behaviors to avoid social activities.

Notably, White’s heuristic model suggests that social isolation could result from incorrect self-perception, such as negative self-evaluation and unrealistic expectations. Furthermore, this model can enable nurses and physicians to analyze the internal influencing factors of social isolation among patients. Moreover, it suggests that nurses and physicians ought to predictably guide the establishment of improved self-cognition while providing psychological care to patients with cancer. It is imperative to address negative self-assessments among patients as soon as they exhibit social isolation behavior. Social isolation is a multidimensional experience, and its antecedents may comprise the aforementioned attributes; thus, future studies must provide an in-depth examination of the relationships among these attributes.

Identifying the attributes of social isolation in adults with cancer has profound implications. Along with the common attributes (i.e., limitations in social networks and feelings of loneliness), living an abnormal life and concealing individual emotions have been identified as characteristics of behaviors and states of social avoidance. The findings from this analysis illustrate the unique aspect of social isolation associated with cancer. Upon our review (Miaskowski et al., 2020; Morreale et al., 2020; Takemura et al., 2021), we also found that the measurement tools used to assess social isolation vary across studies. This confirms a lack of consensus on the concept of social isolation regarding cancer populations. Furthermore, it suggests that future research ought to explore and implement social isolation assessment tools that can perform standardized scoring to facilitate nursing assessment, as well as increase the comparability of this indicator in the cancer population. In future research, antecedent social health factors such as COVID-19 could enhance the complexity associated with the concept of social isolation. Thus, researchers should consider the issue more comprehensively.

### Limitations

Several limitations and biases have been identified in this analysis, including the interpretations of the results and the possibility of an incomplete search of sources. Despite following the guidance of the inductive process underscored in Rodgers’ evolutionary method (Rodgers, 2000), this analysis might contain bias. In an attempt to mitigate this, the process of data extraction was performed independently by two reviewers, and a third reviewer was consulted in case of a disagreement. While a systematic search was conducted, it is unlikely that it was comprehensive. Moreover, this concept analysis only included literature published in English and Chinese, as there is a more extensive volume of articles in these two languages.

## Conclusion

The findings from this concept analysis contribute to the understanding and clarity of social isolation regarding adults with cancer. This analysis could be applied to developing cancer-specific assessment tools and measures that can facilitate early recognition of the requirements underlying psychosocial support and establish a conceptual foundation to mitigate social isolation regarding clinical practice and research. Subjective, perceived social isolation is of specific concern. Further studies are required to examine the relationships among attributes. Moreover, research mitigating social isolation is needed to assist people living with cancer to improve ways of adapting to adversity throughout their experience.

## Author contributions

YL: conceptualization, methodology–data collection, methodology–data analysis, writing–original draft preparation, and writing–reviewing and editing. GH: resources and revision. MW: methodology–data collection and methodology–data analysis. LH: writing–review and editing and supervision. All authors contributed to the article and approved the submitted version.

## Funding

This work was supported by the Shanghai Association For Science and Tech (21002411300) and Innovation research team of high-level local universities in Shanghai (SHSMU-ZDCX20212802).

## Conflict of interest

The authors declare that the research was conducted in the absence of any commercial or financial relationships that could be construed as a potential conflict of interest.

## Publisher’s note

All claims expressed in this article are solely those of the authors and do not necessarily represent those of their affiliated organizations, or those of the publisher, the editors and the reviewers. Any product that may be evaluated in this article, or claim that may be made by its manufacturer, is not guaranteed or endorsed by the publisher.
